# Conditioning with rabbit versus horse ATG dramatically alters clinical outcomes in identical twins with severe aplastic anemia transplanted with the same allogeneic donor

**DOI:** 10.1186/s13045-015-0173-x

**Published:** 2015-06-26

**Authors:** P. T. Vo, J. Pantin, C. Ramos, L. Cook, E. Cho, R. Kurlander, H. Khuu, J. Barrett, S. Leitman, R. W. Childs

**Affiliations:** Hematology Branch, National Heart Lung and Blood Institute (NHLBI), National Institute of Health (NIH), Bethesda, MD USA; Department of Hematology and Oncology, Georgia Regents University, Augusta, GA USA; Department of Clinical Pathology, Hematology Services, National Heart Lung and Blood Institute (NHLBI), National Institute of Health (NIH), Bethesda, MD USA; Cell Lab Processing Section, Department of Transfusion Medicine, National Institute of Health (NIH) Blood Bank, Bethesda, MD USA; Stem Cell Allogenic Transplantation, Hematology Branch, National Heart Lung and Blood Institute (NHLBI), National Institute of Health (NIH), Bethesda, MD USA; Department of Transfusion Medicine, National Heart Lung and Blood Institute (NHLBI), National Institute of Health (NIH), Bethesda, MD USA; Transplantation Immunotherapy, Hematology Branch, National Heart Lung and Blood Institute (NHLBI), National Institute of Health (NIH), Bethesda, MD USA

**Keywords:** Severe aplastic anemia, Rabbit versus horse ATG conditioning, Allogeneic bone marrow transplant, Identical twins

## Abstract

Severe aplastic anemia (SAA) is a rare disorder leading to bone marrow failure, which if left untreated, is invariably fatal. Conventional therapies with immunosuppressive therapy or allogeneic hematopoietic stem cell transplantation (HSCT) are highly effective. HSCT can offer a greater outcome in younger patients who have an available HLA match-related donor. Recent studies showing the addition of antithymocyte globulin (ATG) to the conditioning regimen improves engraftment and reduces the risk of graft-versus-host disease (GVHD).There are currently two ATG preparations in the USA, equine (or horse) and rabbit ATG. These agents are pharmacologically distinct, having significant differences in their pharmacokinetics and in vivo immunosuppressive effects [N Engl J Med 365(5):430–438, 2011]. Here, we report a case of two monozygotic twins with constitutional SAA that evolved to myelodysplastic syndrome (MDS) who both underwent allogeneic peripheral blood stem cell transplantation (PBSC) from the same single HLA antigen mismatched sibling donor with the only difference in the transplant regimen being the type of ATG used in the preparative regimen; one twin received horse ATG and the other received rabbit ATG during conditioning. This report emphasizes that dramatic differences in donor T cell chimerism and clinical outcomes including GVHD can occur as a consequence of the type of ATG that is utilized in the transplant conditioning regimen. These differences highlight that these agents should not be considered interchangeable drugs when used in this setting.

## Background

Severe aplastic anemia (SAA) is a rare disorder leading to bone marrow failure which, if left untreated, is invariably fatal. Annually, 300 to 600 new cases of SAA are diagnosed in the United States of America [1]. Conventional therapies are highly effective and involve either immunosuppressive therapy with horse antithymocyte globulin (ATG) and cyclosporine (CSA) or allogeneic hematopoietic stem cell transplantation (HSCT), with the latter treatment offering superior outcome in younger individuals who have an available human leukocyte antigens (HLA) match-related donor. Significant advances in the procedure have been made over the past three decades that have improved the survival of SAA patients undergoing transplantation. Recent studies have shown that chronic graft-versus-host disease (cGVHD) is reduced when bone marrow as opposed to peripheral blood stem cell transplantation (PBSC) is used as a graft source for SAA. Nevertheless, graft rejection continues to complicate this approach and remains a major contributor to transplant-related mortality. Transplantation of PBSC mobilized from donors using granulocyte-colony stimulating factor (G-CSF) can also be used to cure patients with ATG-refractory SAA and may be associated with a reduced risk of graft rejection, particularly in heavily transfused patients who are at high risk for this complication. Traditionally, high-dose cyclophosphamide has been used to condition SAA patients undergoing transplantation, with recent studies showing that the addition of ATG to the conditioning regimen improves engraftment and reduces the risk of graft-versus-host disease (GVHD). ATG improves engraftment by killing recipient lymphocytes that mediate graft rejection and may also remain in the circulation at the time of the transplant, killing alloreactive donor T cells that mediate GVHD [2–10]. Equine or horse ATG (Atgam) and rabbit ATG (thymoglobulin) are two antithymocyte globulin preparations that are available in the United States of America, for clinical use as immunosuppressive agents. Both drugs have been used in off-label applications as conditioning agents for allogeneic HSCT for a variety of disorders including SAA. However, these agents are pharmacologically distinct, having significant differences in their pharmacokinetics and in vivo immunosupressive effects. A recent randomized trial comparing these two agents as upfront therapy for SAA outside of the context of a transplant showed the depth and duration of CD3 T cell lymphopenia was greater with rabbit ATG compared to equine ATG. These differences, as well as the observation that responses in SAA were greater with equine ATG compared to rabbit ATG, highlight that these agents should not be considered interchangeable drugs when used in this setting [2].

## Case report

Twin #1 and twin #2 are two Vietnamese female monozygotic twins who were born prematurely at 8 months of gestation with a birth weight of approximately 1.8 kg each. They were both diagnosed with SAA at 2 years of age when they presented with symptoms related to pancytopenia. Their initial treatment in Vietnam consisted of corticosteroids and RBC and platelet transfusion support. In October 2001, at the age 8, both twins were referred to the NHLBI in the United States of America, for further evaluation and management.

**Fig. 1:**
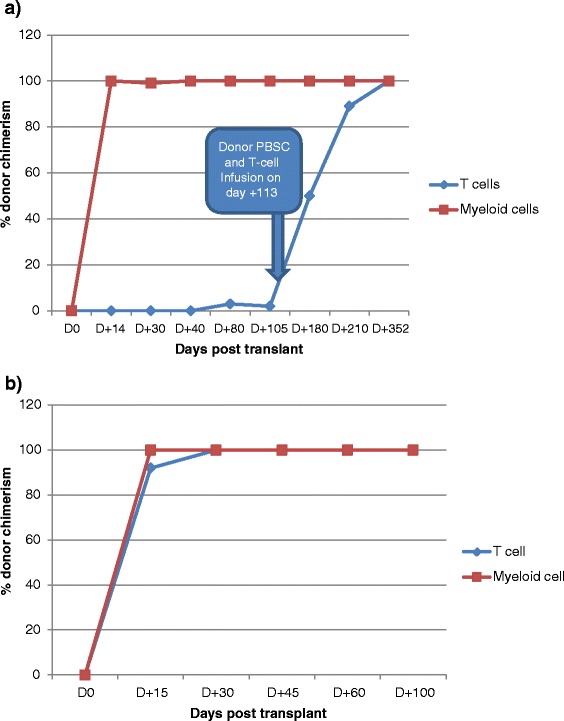
**a** Twin #1 chimerism timeline. **b** Twin #2 chimerism timeline

Twin #1 was the first of the two identical twins to be diagnosed with SAA and the first to develop myelodysplastic syndrome (MDS). A bone marrow biopsy in October 2001 showed 5–50 % cellularity without evidence of dysplasia or blasts but with trisomy 8 being present in 35 % of metaphases consistent SAA evolution to MDS. Testing for Fanconi’s anemia and paroxysmal nocturnal hemoglobinuria (PNH) were negative. Peripheral blood counts demonstrated an absolute neutrophil count (ANC) of 417 cells/μl, a platelet count of 16 k/μl, hemoglobin of 10 g/dl, and an absolute reticulocyte count of 61 k/μl. She failed a trial of danazol therapy and in April 2002, commenced treatment with horse ATG 40 mg/kg/day for 4 days combined with CSA. Her post-immunosuppression course was complicated by multiple episodes of infection including non-specific ileitis, colitis, bronchitis, pharyngitis, pneumonia, and an ulcerative labial infection. There was no response at 6 months to horse ATG and she remained transfusion dependent. In November 2002 at age 9, she underwent an allogeneic PBSC transplant from her 9/10 HLA match-related brother (donor and patient mismatched at a single HLA A loci and fully matched at HLA at the HLA B, C, DR, and DQ loci) after conditioning with fludarabine 25 mg/m^2^ × 5 days, cyclophosphamide 60 mg/kg × 2 days, and rabbit ATG 3.5 mg/kg/day × 4 days. CSA and MTX were used as GVHD prophylaxis. She had neutrophil recovery on day +15 and platelet recovery on day +9. Her post-transplant course was complicated by CMV reactivation which responded to antiviral therapy and bacterial sepsis which resolved with IV antibiotics. Lineage-specific chimerism studies conducted on multiple occasions from the time of engraftment until day +100 revealed extremely low levels of donor T cell engraftment. After day 100, she was observed to have a rapid decline in her absolute reticulocyte count, which in the context of very low levels of donor T cell chimerism, led to concerns of impending graft rejection. Therefore, on transplant day +113, she received an intravenous infusion of G-CSF-mobilized PBSCs from her original stem cell donor containing 2 × 10^6^ CD34+ cells/kg and 1.4 × 10^8^ CD3+/kg cells. She eventually converted 100 % donor T cell and myeloid chimerism by transplant day +352 (Fig. 1a). She never developed acute GVHD but did develop limited mouth and skin chronic GVHD on day +175. By 6 years post-transplant, her mild oral GVHD had completely resolved and all immunosuppressive medications were discontinued.

Twin #2 was the second twin diagnosed with aplastic anemia. On presentation to the NHLBI in 2002, she had an ANC of 2100 cells/μl, a platelet count of 45 k/μl, a hemoglobin of 13.7 g/dl, and an absolute reticulocyte count of 80.7 k/μl. Her bone marrow biopsy demonstrated hypocellular marrow with less than 5 % cellularity with a marked decrease in megakaryocytes. At that time, chromosome analysis by metaphase banding of the peripheral blood and skin fibroblasts revealed no evidence of trisomy 8 or any other chromosome abnormality. Testing for Fanconi’s anemia and PNH was negative. Similar to her sister, she failed a 3-month trial of danazol treatment. Two years later, in January 2003, she had a decline in her blood counts: her ANC declined to 996/μl, platelets to 15 k/μl, and hemoglobin to 9.4 g/dl, with an absolute reticulocyte count of 74.2 k/μl. A repeat bone marrow biopsy performed in 2004 revealed 5–15 % cellularity with trisomy 8 now being detectable in 13 % of metaphases consistent with evolution to MDS. In April 2004 at the age 11, she underwent an allogeneic PBSC transplant from her 9/10 HLA match-related brother who had donated PBSCs for her syngeneic twin who had been transplanted 1.5 years earlier (similar to twin #1, the donor and twin #2 were mismatched at a single HLA A loci and fully matched at HLA at the HLA B, C, DR, and DQ loci). Of note, both twins received similar transplanted allograft doses of CD34+ cells/kg and T cells/kg (Table 1). Twin #2’s transplant conditioning regimen was virtually identical to her twin sister except horse ATG (40 mg/kg/day × 4 days) rather than rabbit ATG was utilized, given concerns that the latter drug may have contributed to her twin sister’s poor donor T cell engraftment. She had sustained donor engraftment with neutrophil recovery occurring on day +12. In contrast to her sister, she achieved very rapid donor T cell engraftment, demonstrating 100 % donor T cell chimerism by day +30 (Fig. 1b). Her post-transplant course was complicated by grade II acute GVHD involving her gut which responded to corticosteroid treatment. At 6 months post-transplant, she developed extensive chronic GVHD of the skin with a lichenoid rash on her face, trunk, and extremities that improved partially following treatment with rituximab. At 9 months post-transplant, she also developed chronic GVHD of the eyes treated with topical corticosteroids and placement of silicone punctal plugs. At 13 months post-transplant, she was diagnosed with BOS which was treated with systemic and inhaled corticosteroids and later with inhaled cyclosporine solution [11]. Although her cGVHD symptoms improved over time, she is now 10+ years post-transplant and, in contrast to her identical twin sister, continues to require immunosuppressive therapy with prednisone, MMF, and tacrolimus to treat her chronic skin GVHD (Fig. 3a–d).

**Table 1:** Comparison of clinical data

Clinical data	Twin #1	Twin #2
Disease progression		
Age of presentation	2	2
Age at transplantation (years)	9	11
Sex	Female	Female
Diagnosis	Constitutional SAA with trisomy 8	Constitutional SAA with trisomy 8
Karyotype at transplant	Trisomy 8	Trisomy 8
Pre-transplantation characteristics		
Absolute neutrophil count (k/μl)	417	996
Absolute reticulocyte count(k/μl)	61	71.2
Platelets (k/μl)	16	15
Time from diagnose to PBSC (years)	7 years	9 years
Recipient blood type	AB+	AB+
Donor relationship	Brother	Brother
Donor blood type	A+	A+
Donor sex	Male	Male
HLA match	9 out of 10	9 out of 10
RIC allogeneic PBSC		
Age at transplant	9	11
Recipient blood type	AB+	AB+
CD34+ cell dose (×10^6^/kg)	6.24 × 10.6	6.32 × 10.6
CD3+ cell dose (×10^7^/kg)	42.3 × 10.7	35.2 × 10.7
Day of 100 % donor T cell chimerism	Day +352	Day +30
Received DLI and stem cell boost	Yes	No
Prophylaxis against GVHD	MTX 5 mg/m^2^ day +1, +3, +6	MTX 5 mg/m^2^ day +1,+3, +6
	Cyclosporine	Cyclosporine
Conditioning regimen comparison		
Cyclophosphamide 60 mg/kg IV × 2	Yes	Yes
Fludarabine 25 mg/m^2^ IVBP × 5	Yes	Yes
Rabbit ATG 3.5 mg/kg IV × 4	Yes	No
Horse ATG 40 mg/kg IV × 4	No	Yes
GVHD prophylaxis		
MTX 5 mg/m^2^ × 3	Yes	Yes
Cyclosporine	Yes	Yes
Post-transplantation GVHD		
Time from HSCT to GVHD (months)	20 months	<1 month
Acute GVHD	No	Yes (grade II GI tract)
Site of cGVHD	Skin, mouth	Skin, ocular, and lung
cGVHD grade (severity)	Limited	Extensive
cGVHD treatment	Daclizumab, cyclosporine	Cyclosporine, sirolimus, MMF, steroid
		Daclizumab, rituximab, tacrolimus

## Discussion

Both immunosuppressive therapy and allogeneic hematopoietic stem cell transplantation represent effective therapy for patients with SAA [9, 10, 12, 13]. Graft rejection and GVHD, both which are critically influenced by the composition of the allograft and the agents used in the preparative regimen, remain obstacles to the success of stem cell transplantation. Although donor T cells are the main mediators of acute GVHD, their engraftment is critical to prevent graft rejection and to restore protective host immunity post-transplant. Research continues to determine the optimal conditioning regimen that promotes donor engraftment while avoiding GVHD. Previously, we have shown that a transplant regimen utilizing cyclophosphamide, fludarabine, and equine ATG conditioning with a G-CSF-mobilized PBSC transplant results in excellent engraftment and survival in heavily transfused SAA patients who have failed prior immunosuppressive therapy. However, this regimen often results in very rapid donor T cell engraftment, which was recently shown to be an independent variable in a multivariate analysis increasing the risk of chronic GVHD [14].

**Fig. 2:**
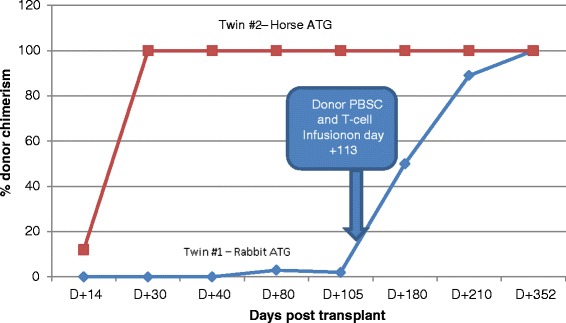
Comparison of T cell chimerism between twins

**Fig. 3:**
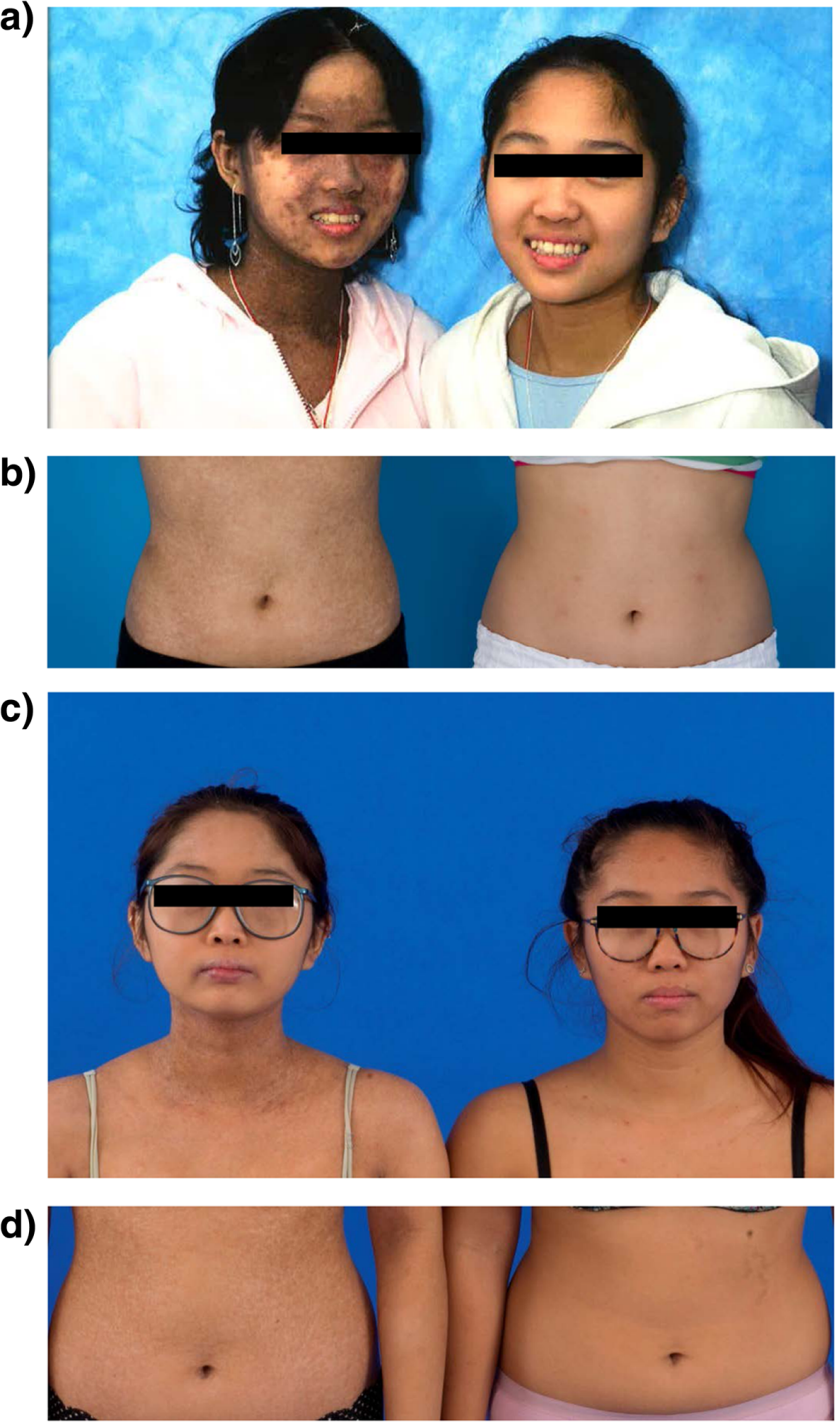
**a, b** Twin #2 (left) 5 years and 3 months post-transplant and twin #1 (right) 6 years and 8 months post-transplant. **c**, **d** Twin #2 (left) 10 years and 6 months post-transplant and twin #1 (right) 11 years and 11 months post-transplant

Here, we report data where syngeneic twins with MDS who underwent allogeneic transplantation from the same donor had dramatically different donor T cell engraftment profiles and transplant outcome as a consequence of using rabbit versus horse ATG in the preparative regimen (Fig. 2). Twin #1, who received rabbit ATG with her conditioning, had a post-transplant course which was associated with exceedingly low degrees of donor T cell engraftment and a declining reticulocyte count necessitating a subsequent infusion of donor PBSCs and T cells to avoid graft rejection. Remarkably, this patient did not achieve full donor T cell chimerism until day +352, never developed acute GVHD, and only experienced mild chronic oral GVHD which occurred after the PBSC boost. In contrast, twin #2 who received from the same donor virtually the identical number of transplanted donor CD34+ cells and a slightly lower number of transplanted CD3+ T cells, received horse ATG with the conditioning regimen and experienced very rapid and complete donor T cell chimerism which was associated with both acute GVHD and extensive chronic GVHD (Fig. 3a–d). Of note, twin #1 but not twin #2 had received ATG previously before the transplant, and the donor and twin #2 were 2 years older when the transplant was performed on twin #2 relative to twin #1. Therefore, we are unable to conclude with complete certainty that the differences in clinical outcomes were solely related to the use of rabbit versus horse ATG in the conditioning regime. However, it is important to note that because twin #1 but not twin #2 had received ATG previously before the transplant as treatment for her aplastic anemia, she would have been expected to be the more immunosuppressed twin pre-transplant. This makes the chimerism differences more likely attributable to the type of ATG utilized during conditioning, as one would have expected to the more immunosuppressed twin to have faster donor T cell engraftment, which was not the case with twin #1. We theorize that the differences in the half-life of ATG likely accounted for the dramatic differences in the speed of donor T cell engraftment and the occurrence of and severity of GVHD observed between these patients. Rabbit ATG has a much longer half-life (29.8 days) compared to horse ATG (5.7 days) [15, 16]. As a consequence, rabbit ATG likely depleted transplanted donor T cells in vivo much more efficiently in twin #1 compared to twin #2 (who received horse ATG), which prevented acute GVHD but also dramatically delayed donor T cell engraftment, placing the patient at increased risk for graft rejection. Given its shorter half-life, horse ATG given to twin #2 was less effective in inducing in vivo T cell depletion of transplanted donor cells, which resulted in very rapid donor T cell engraftment with subsequent acute and chronic GVHD. A recent retrospective, non-randomized study comparing horse to rabbit ATG in addition to cyclophosphamide as conditioning for allogeneic HSCT patients with SAA reported similar observations, namely conditioning with rabbit ATG was more protective against acute and chronic GVHD than conditioning with horse ATG, with rabbit ATG being associated with a higher incidence of mixed chimerism. Recipients of rabbit ATG were also observed to have a higher incidence of viral and fungal infections [17].

Outside of the setting of a transplant, initial treatment of SAA with horse ATG has shown to result in higher responses rates than rabbit ATG [2]. However, rabbit ATG is effective as salvage therapy for patients with SAA who have disease that is refractory or has relapsed after initial therapy with horse ATG [2, 18, 19]. These findings suggest that differences in the pharmacokinetics of these agents alone do not fully account for differences in the biological activity of these agents in patients with SAA. Recent data have shown that rabbit ATG depletes different lymphocyte subsets compared to horse ATG, and rabbit ATG is more likely to promote the expansion of CD4 + CD25 bright FOXP3+ regulatory T cells [2, 20]. Taken altogether, these data establish rabbit and horse ATG as having distinct in vitro and in vivo immunosuppressive properties that should be considered when selecting the formulation of ATG to be utilized in the transplant conditioning regimen.

## Conclusion

In this paired case report, we demonstrate the unique experience of two monozygotic patients with bone marrow failure and MDS who received an allogeneic HPSC transplant from the same donor, with the only difference in the transplant regimen being the formulation of ATG used in the preparative regimen. Remarkably, their T cell engraftment profiles and clinical outcomes including acute and chronic GVHD differed dramatically as a consequence of this difference.

Rabbit ATG resulted in superior GVHD prophylaxis in twin #1, which came at the cost of impeding donor T cell engraftment placing the patient at risk for graft rejection. This case highlights that rabbit ATG and horse ATG should not be considered interchangeable drugs, with the need to consider these effects before choosing the type of ATG to be used in the preparative regimen. In the risk where graft rejection is low, one might consider that rabbit ATG might be better suited to be utilized as part of conditioning regimen, with salvage donor lymphocyte infusions being incorporated into transplant regimens to prevent graft rejection in patients with falling or low degrees of donor T cell chimerism. In situations where the risk of graft rejection or failure is high, given its much shorter half-life, the use of horse ATG may have a role in helping to eradicate recipient immunity without leading to significant in vivo depletion of donor T cells. In contrast, in scenarios where the risk of GVHD may be high (i.e., HLA mismatched transplants), the use of rabbit ATG in the preparative regimen may be more appropriate and gives its greater ability to lead to in vivo donor T cell depletion which reduces the risk of GVHD.

## Consent

Informed consent to publish the information was granted from the patients or their guardians.

## Abbreviations

ANC: absolute neutrophil count
ATG: antithymocyte globulin
BOS: bronchiolitis obliterans syndromes
CMV: cytomegalovirus
CSA: cyclosporine
cGVHD: chronic graft-versus-host disease
DLI: donor lymphocyte infusion
G-CSF: granulocyte-colony stimulating factor
GVHD: graft-versus-host disease
HLA: human leukocyte antigens
HSCT: hematopoietic stem cell transplantation
MDS: myelodysplastic syndrome
MMF: mycophenolate mofetil
MTX: methotrexate
NHLBI: National Heart Lung and Blood Institute
PBSC: peripheral blood stem cell transplantation
PNH: paroxysmal nocturnal hemoglobinuria
SAA: severe aplastic anemia
